# Dosimetric comparison of postoperative interstitial high-dose-rate brachytherapy and modern external beam radiotherapy modalities in tongue and floor of the mouth tumours in terms of doses to critical organs

**DOI:** 10.2478/raon-2023-0050

**Published:** 2023-11-30

**Authors:** Örs Ferenczi, Tibor Major, Georgina Fröhlich, Dalma Béla, Szabolcs Tódor, Csaba Polgár, Hironori Akiyama, Botond Bukovszky, Zoltán Takácsi-Nagy

**Affiliations:** Centre of Radiotherapy, National Institute of Oncology, Budapest, Hungary; Department of Oncology, Semmelweis University, Budapest, Hungary; National Tumour Biology Laboratory, National Institute of Oncology, Budapest, Hungary; Eötvös Loránd University, Faculty of Science, Budapest, Hungary; Department of Oral Radiology, Osaka Dental University, Osaka, Japan; Department of Oral Diagnostics, Semmelweis University, Budapest, Hungary

**Keywords:** floor of mouth tumour, tongue tumour, HDR, brachytherapy, VMAT, stereotactic, dosimetry, cyberknife

## Abstract

**Background:**

The aim of the study was to dosimetrically compare interstitial high-dose-rate (HDR) brachytherapy (BT) and modern external beam radiotherapy modalities, as volumetric modulated arc therapy (VMAT) and stereotactic radiotherapy with Cyberknife (CK) of tumours of the tongue and floor of the mouth in terms of dose to the critical organs.

**Patients and methods:**

In National Institute of Oncology, Budapest, between March 2013 and August 2022 twenty patients (11 male/9 female) with stage T1–3N0M0 tongue (n = 14) and floor of mouth (n = 6) tumours received postoperative radiotherapy because of close/positive surgical margin and/or lymphovascular and/or perineural invasion. High-dose-rate interstitial brachytherapy applying flexible plastic catheters with a total dose of 15 × 3 Gy was used for treatment. In addition to BT plans VMAT and stereotactic CK plans were also made in all cases, using the same fractionation scheme and dose prescription. As for the organs at risk, the doses to the mandible, the ipsilateral and the contralateral salivary glands were compared.

**Results:**

The mean volume of the planning target volume (PTV) was 12.5 cm^3^, 26.5 cm^3^ and 17.5 cm^3^ in BT, VMAT and CK techniques, respectively, due to different safety margin protocols. The dose to the mandible was the most favourable with BT, as for the salivary glands (parotid and submandibular) the CK technique resulted in the lowest dose. The highest dose to the critical organs was observed with the VMAT technique. The mean values of D_2cm_3 and D_0.1cm_3 for the critical organs were as follows for BT, VMAT and CK plans: 47.4% and 73.9%, 92.2% and 101.8%, 68.4% and 92.3% for the mandible, 4.8% and 6.7%, 7.3% and 13.8%, 2.3% and 5.1% for the ipsilateral parotid gland, 3.5% and 4.9%, 6.8% and 10.9%, 1.5% and 3.3% for the contralateral parotid gland, 7.3% and 9.4%, 9.0% and 14.3%, 3.6% and 5.6% for the contralateral submandibular gland.

**Conclusions:**

The present results confirm that BT, despite being an invasive technique, is dosimetrically clearly beneficial in the treatment of oral cavity tumours and is a modality worth considering when applying radiotherapy, not only as definitive treatment, but also postoperatively. The use of the CK in the head and neck region requires further investigation.

## Introduction

Surgery is usually the primary treatment for advanced tumours of the oral cavity, including the tongue and the floor of the mouth, but smaller lesions can also be treated with laser resection, cryotherapy, external beam radiotherapy (EBRT) or brachytherapy (BT). The latter is particularly important in the treatment of early-stage oral malignant lesions.^1,2,3,4^ If surgery is performed for small tumours (T1–2), postoperative irradiation may be necessary based on the surgical histology (close or positive surgical margins, lymphovascular or perineural invasion).^5,6^ If the neck staging shows no regional metastasis and the depth of tumour invasion is less than 5 mm, treatment of the neck with either dissection or RT is not necessary.^7,8^ In such early-stage lesions, postoperative radiotherapy can be performed using either percutaneous or interstitial RT. The aim of RT is to administer the maximum dose to the target volume (tumour bed). However, with external RT unnecessary radiation exposure to the surrounding critical organs (salivary glands, mandible, spinal cord, *etc*.) may result, thereby increasing the incidence of side effects (xerostomia, osteoradionecrosis, fibrosis, trismus, *etc*.).

Today, the state-of-the-art irradiation modality routinely used is intensity modulated radiation therapy (IMRT), where a multileaf collimator (MLC) allows accurate tracking of the three-dimensional (3D) shape of the target volume using a reference isodose surface, while significantly reducing exposure of critical organs.^9,10^ An improved version of this is rotating-arc intensity modulated radiotherapy or volumetric modulated arc therapy (VMAT), which allows even more precise irradiation of very complex target volumes (*e.g.* head and neck tumour regions) while further reducing the dose burden on the tissues to be protected.^11,12^

The current flagship of stereotactic RT is the Cyberknife (CK) technique. The treatment aims to deliver the highest possible dose to the tumour using many non-coplanar beams. At the same time, the surrounding healthy tissue receives a relatively low dose and remains intact as the beams are scattered in a 3D geometry.^13,14^

The tumour bed can also be treated with interstitial BT for oral cavity tumours, if indicated.^2,15,16^ The treatment involves introducing radioactive isotope(s) into the tissue of the target volume by direct implantation (seed) or by applicators (rigid metal needles, flexible plastic catheters). BT allows a higher dose to be delivered locally and provides greater protection to surrounding intact tissue due to the rapid dose fall-off around the source. Alongside the long-established low dose rate (LDR) BT, the increasingly widely used high dose-rate (HDR) method can produce the same therapeutic results, but while the former requires better patient cooperation due to the need for isolation and longer treatment times, the latter method eliminates these problems.^17,18^

In the current model study, we compared HDR postoperative BT plans of 20 patients treated with tongue and floor of mouth cancer with VMAT and CK treatment plans in terms of dosimetry of the organs at risk (OARs).

## Patients and methods

At National Institute of Oncology, Budapest, between January 2016 and December 2021, 20 patients (T1–3N0) underwent tumour extirpation and unilateral (85%, 17/20) or bilateral (15%, 3/20), selective neck dissection for tongue or sublingual cancers following negative neck staging. Histology did not confirm metastatic lymph node. For local postoperative BT to be justified, one of the following criteria had to be met: T3 tumour, surgical margin ≤ 2 mm, lymphovascular infiltration or perineural invasion. Based on the processing of histopathology, 20% had T3 size (TNM 8th)^19^, 85% had a surgical margin of ≤ 2 mm and 40% had perineural spread. The treatments were performed with an HDR afterloading device using Iridium-192 isotope (Flexitron, Elekta Brachytherapy, Veenendaal, The Netherlands) after implantation of flexible catheters (median 6, range 6–8) into the tumour bed. The insertion was performed via submental penetration by the help of trocars, in the operating room, under general anaesthesia. The mean time between interstitial BT (implantation) and surgery was 54 days (range: 42–81 days).

### Brachytherapy planning

After catheter implantation, all patients underwent CT imaging with 3 mm slice thickness covering the whole head including the tumour bed, the parotids and submandibular gland. In all cases, BT planning with Oncentra Brachy v4.5.3 (Elekta Brachytherapy, Veenendaal, The Netherlands) was performed. The total dose of BT was 45 Gy. 3 Gy per fraction was delivered twice daily, 6 hours apart. Imaging of the primary tumour (CT, magnetic resonance imaging [MRI]) and palpation of the surgical site helped to determine the target volume (clinical target volume [CTV]: tumour bed [gross tumor volume, GTV] + 0.5 cm safety margin). There was no safety margin around the CTV, so the planning target volume (PTV) was equal to the CTV. The ipsilateral (il.) and contralateral (cl.) parotids and cl. submandibular salivary gland, the skin and the mandible were contoured as organs to be protected. Skin was defined as a layer of 0.5 cm below the outer body surface. Source dwell positions and dose reference points were determined individually for each implant. Geometric and graphical dose optimization was performed. The isodose line for dose prescription was chosen to achieve 90% dose coverage of the PTV (V100 = 90%). The BT planning was based on the recommendations of GEC-ESTRO (Groupe Européen de Curiethérapie and the European Society for Radiotherapy and Oncology) Head and Neck Working Group.

### VMAT planning

To prepare the external beam RT plan, the CT images of the patients were exported to the external planning system (Eclipse v11, Varian, USA) complying with the DICOM (Digital Imaging and Communications in Medicine) RT protocol together with the structure set defined in BT plans, and subsequently the IMAT plans were prepared. This method ensured that the target volume and the organs to be protected were always exactly the same in the two planning systems, thus eliminating inaccuracies due to contouring. From this it also follows that the differences obtained in the comparison were due solely to the differences between the two irradiation techniques and were not influenced by other factors. For the IMAT plans, the CTV was extended by 3 mm in each direction to create the PTV. The VMAT plans were created using 6 MV photon energy. VMAT plans were optimized using the Varian RapidArc progressive resolution optimization algorithm (PRO) and the dose was calculated using the analytical anisotropic algorithm (AAA). After dose normalization the coverage of the PTV by the prescribed dose (PD) was 90% (V100 = 90%).

### Cyberknife planning

In order to prepare the stereotactic plans, the CT images and the RT structures (Radiotherapy Structure Set) were transferred from the Eclipse system to the Accuray Precision (Accuray, Sunnyvale, CA, USA) version 3.1.0.0. planning system. The PTV used for stereotaxic plans was created by extending the brachytherapy CTV symmetrically with 2 mm. The Cyberknife plans were generated using the multileaf collimator system, 6MV FFF photon energy using the VOLO optimizer for dose optimization and the FSPB (Finite Size Pencil Beam) for dose calculation. The dose prescription was chosen to achieve V100 = 90% for the PTV.

### Comparison of the plans

The same dose prescription and fractionation (15 × 3 Gy) were used for all three techniques. Parameters calculated from dose volume histogram were used to compare the plans. To describe the target coverage, the volume of the PTV irradiated by the PD was used (V100). The objective comparison was based on the same target coverage, V100 = 90%, for all three techniques. It follows from this that any differences found between the plans were only due to the characteristics of the irradiation techniques. The conformity of dose distributions was quantified using the conformal index (COIN), which takes into account both the target coverage and the unnecessary irradiation of normal tissues.^20^ Its maximum value is 1, and the higher the value, the more conformal the dose distribution. Dose homogeneity was characterized with the dose nonuniformity ratio (DNR) in BT plans, and homogeneity index (HI) in the VMAT and CK plans. DNR is the ratio of volume irradiated by 1.5 times the PD to volume irradiated by the PD. The HI was calculated according to recommendation of ICRU (International Commission on Radiation Units and Measurements) Report 83.^21^ By definition, HI = (D2%–D98%)/D50%. To characterize the unintended irradiation of OARs, small volumes of high dose were used. D_xcm_3 represents the minimum dose to the most exposed × cm^3^ of an organ (mandible, parotid). For all OARs mean D_2cm_3 and D_0.1cm_3 were calculated and compared.

Friedman ANOVA and Fisher-LSD (Least Significant Difference) post-hoc tests were used (Statistica 12.5, StatSoft, Tulsa, OK, USA) to compare dose volume parameters of VMAT, CK and HDR BT techniques. The level of significance was 0.05.

## Results

**FIGURE 1.**
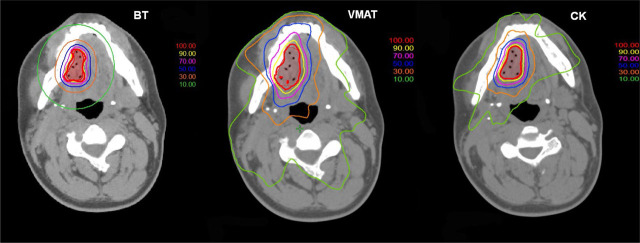
Representative dose distributions in a brachytherapy (BT), a volumetric modulated arc therapy (VMAT) and a Cyberknife (CK) plan.

**FIGURE 2.**
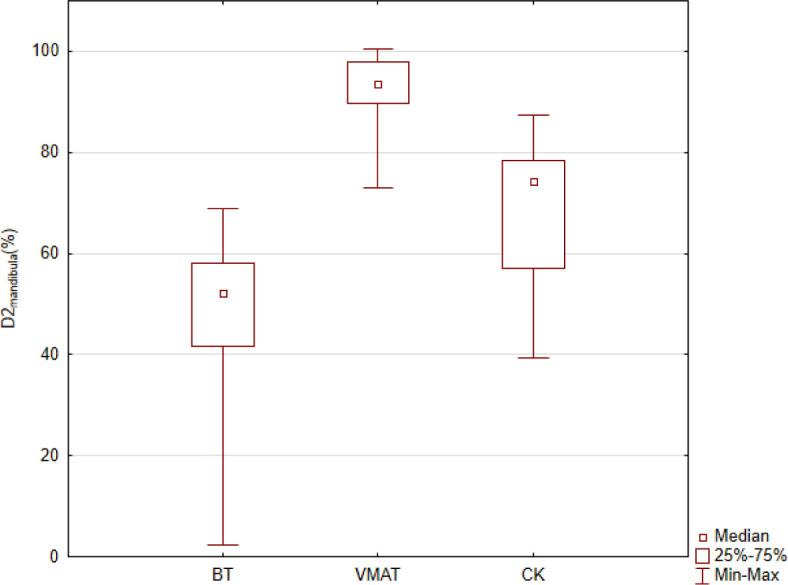
Mean dose in % to the most exposed 2 cm^3^ volume of the mandible. BT = brachytherapy; CK = Cyberknife; VMAT = volumetric modulated arc therapy

**FIGURE 3.**
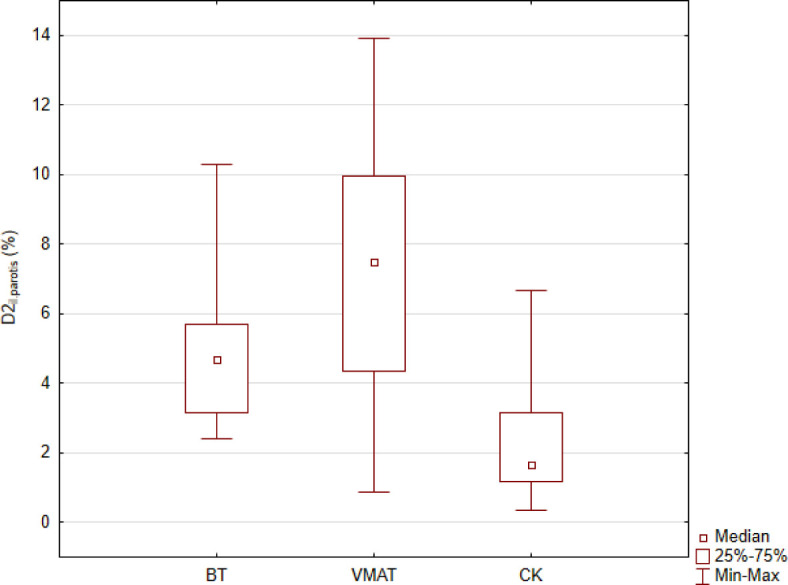
Mean dose to the most exposed 2 cm^3^ volume of the ipsilateral parotid. BT = brachytherapy; CK = Cyberknife; VMAT = volumetric modulated arc therapy

Due to the same dose prescription (V100 = 90%) the mean target volume dose coverage in all modalities was 90.0%. Figure 1 shows representative dose distributions for the three investigated techniques. It can be seen that the target was irradiated properly in each case, but notable differences can be observed for the volumes irradiated by doses corresponding to middle and lower isodose values (< 70%). In the BT plan, these volumes are the smallest, especially in regions near the target volume. Table 1 shows the dosimetric data for PTV. Due to the safety margins used in VMAT and CK plans, the largest volume was for VMAT and the smallest for BT. The plans were more conformal with EBRT compared to BT. The most conformal plans occurred with CK, probably due to the lots of non-coplanar beams. However, the VMAT plans were more homogeneous than the CK plans (HI: 0.09 *vs.* 0.20). It is obvious, that with BT the homogeneity is much worse, and the comparison with EBRT is meaningless. Table 2 shows the quantitative dosimetric parameters for the OARs. The dose to the mandible was the lowest with the use of BT (mean D_2cm_3: 47.4% p < 0,001) compared to the other modalities: VMAT (92.2%) and CK (68.4%). Regarding the salivary glands, the CK technique resulted in the lowest dose on both the ipsilateral and contralateral sides (il. parotid gland, cl. parotid gland, and cl. submandibular gland - CK mean D_2cm_3: 2.3% (p < 0,001), 1.5% (p < 0,001), 3.6% (p < 0,001) *vs.* BT: 4.8%, 3.5%, 7.3% *vs.* VMAT: 7.3%, 6.8%, 9.0%) (Table 1). Similar results were obtained by comparing the values of D_0.1cm_3. The data in Table 2 clearly show that out of the three techniques VMAT resulted in the highest doses to the protected organs. Figures 2 and 3 graphically show the comparisons of D_2cm_3 for the mandible and for the il. parotid gland.

**TABLE 1.** Mean dosimetric parameters of planning target volume (PTV) with ranges

	**BT**	**VMAT**	**CK**	p-value*	BT *vs.* VMAT**	BT *vs.* CK**	VMAT *vs.* CK**
**V_PTV_ (cm^3^)**	12.5 (2.6–21.5)	26.5 (7.7–42.6)	17.5 (5.6–33.6)	< 0.001	< 0.001	0.5553	0.0043
**Conformal index**	0.62 (0.48–0.80)	0.84 (0.78–0.87)	0.86 (0.79–0.93)	< 0.001	< 0.001	< 0.001	0.5480
**Homogenity index**	DNR = 0.38 (0.30–0.50)	0.09 (0.05 – 0.10)	0.20 (0.17–0.20)	NA	NA	NA	< 0.001

* = Friedman ANOVA test;

** = LSD post hoc test

BT = brachytherapy; COIN = conformal index; CK = Cyberknife; DNR = dose non-uniformity ratio; NA = not available; PTV = planning target volume; VMAT = volumetric modulated arc therapy; V_PTV_ = volume of the planning target volume

**TABLE 2.** Mean dosimetric parameters of organs at risk (OARs) with ranges

		**BT**	**VMAT**	**CK**	p-value*	BT *vs*. VMAT**	BT *vs*. CK**	VMAT *vs*. CK**
**Mandible**	D2 (%)	47.4 (29.2–73.4)	92.2 (73.1–100.4)	68.4 (39.3–87.3)	< 0.001	< 0.001	< 0.001	< 0.001
	D0.1 (%)	73.9 (1.7–93.9)	101.8 (97.1–103.9)	92.3 (72.7–100.7)	< 0.001	< 0.001	< 0.001	NS
**Ipsilateral parotid gland**	D2 (%)	4.8 (2.5–11.9)	7.3 (0.9–13.9)	2.3 (0.3–6.7)	< 0.001	0.0011	NS	< 0.001
	D0.1 (%)	6.7 (3.5–19.0)	13.8 (3.7–25.0)	5.1 (0.3–12.3)	< 0.001	< 0.001	NS	< 0.001
**Contralateral parotid gland**	D2 (%)	3. (0.0–7.6)	6.8 (0.6–15.8)	1.5 (0.0–4.7)	< 0.001	0.0018	NS	< 0.001
	D0.1 (%)	4.9 (0.0–11.9)	10.9 (0.9–20.2)	3.3 (0.3–14.0)	< 0.001	0.0105	NS	0.0020
**Contralateral submandibular gland**	D2 (%)	7.3 (3.9–16.3)	9.0 (0.8–17.7)	3.6 (2.0–6.0)	0.0098	NS	0.0198	0.0016
	D0.1 (%)	9.4 (6.2–21.4)	14.3 (2.1–23.1)	5.6 (3.0–11.3)	0.0098	NS	0.0146	< 0.001

* = Friedman ANOVA test;

** = LSD post hoc test

BT = brachytherapy; CK = Cyberknife; DX = dose to the most exposed X cm^3^ volume; NS = non-significant; VMAT = volumetric modulated arc therapy

## Discussion

The comparison of new radiotherapy technologies in the head and neck region has recently become a very interesting area of research. In this study, we performed a dosimetric analysis of 20 cases requiring exclusive postoperative irradiation of the tumour bed. The analysis allowed us to compare our BT planning with VMAT and CK techniques for the same target volume, with special attention to the doses to OARs. In a recent review paper BT was dosimetrically compared to modern EBRT techniques for various cancer types.^22^ Although other author used more fractions with the same dose per fraction (18x3 Gy), we have been using 15x3 Gy fractionation in exclusive postoperative BT since 2014, in line with international recommendations, and our experience so far is that it is well tolerated by patients, with no grade 4 toxicity.^2,15,23,24^

It was shown that from a dosimetric point of view, BT can compete with even the most advanced EBRT techniques, in respect of a higher dose centrally within the target volume and sparing adjacent OARs. However, only a few publications are available in the literature that compare dose-volume parameters of critical organs using BT or other RT modalities.

Sresty *et al.*^25^ compared plans of image guided HDR-BT and IMRT for mobile tongue cancer and found a very good dose conformity in image guided BT (IGBT), which was almost the same as in IMRT, but the dose to the critical structures was lower in BT in all of the cases. Yoshida *et al.*^26^ were the first, who reported dose volume histogram analysis of HDR BT for mobile tongue cancer in 2014. In their five patients - applying image-based planning - the mean V100(CTV), the mean D_0.1cm_3(mandible) and D_2cm_3(mandible) were 98.1%, 80.1% and 55.7%, respectively. Yoshida's results were supported by the work of Akiyama *et al.*^27^ published in 2018. This study is considered to include the largest number of patients in this respect. The study was designed to present dosimetric comparison of IGBT with VMAT for head and neck cancer regarding conformity of dose distribution to PTV and doses to the OARs. Thirty-eight consecutive patients with T1-4 mobile tongue, floor of mouth and base of tongue cancer treated with IGBT were selected. For these patients additional VMAT treatment plans were also prepared using identical CT data. V100 was superior with IGBT (89.0% *vs.* 76.7%, *p* < 0.05). Significantly lower values were obtained with IGBT to OARs compared with VMAT (mandible: D_0.1cm_3 77.0 *vs.* 85.4, D_2cm_3 48.4 *vs.* 68.4, p < 0.05; il. parotid gland: D_0.1cm_3 9.1% *vs.* 13.8%, D_2cm_3 7%, *vs.* 10.5%, p < 0.05; cl. parotid gland: D_0.1cm_3 8.9% *vs.* 15.3%, D_2cm_3 4.9% *vs.* 9.1%, p < 0.05; cl. submandibular gland: D_0.1cm_3 13.4% *vs.* 29.7%, D_2cm_3 8.1% *vs.* 18.3%, p < 0.05). The results prove the superiority of IGBT in the protection of OARs and the important role of this invasive method in the era of new external beam techniques. Similarly, we have currently achieved favourable results with IGBT compared to VMAT in respect of the protection of critical organs. Akiyama and colleagues used the same PTV for BT and VMAT, but we used the extension usually applied for external irradiation (CTV + 3 mm), so the volume of mean PTV for BT was smaller (V_PTV_ 12.5 cm^3^ *vs.* 26.5 cm^3^, p < 0.001), which is also an advantage of this technique, as it is more suitable for protecting the surrounding intact tissue. Of the three techniques, the most conformal dose distributions were obtained with CK (COIN = 0.86), but in contrast, homogeneity was better with VMAT (HI = 0.09). For BT, the conformality was inferior compared to the EBRT, but its advantage was in lower doses to mandible.

Osteoradionecrosis (ORN) of the mandible is one of the most dreaded complications of head and neck irradiation. The incidence of ORN has decreased in recent times, from approximately 20% (several decades ago) to 4–8% (in the modern era). This tendency might be attributed to improvements in RT techniques, such as the IMRT currently used.^28^ Peterson *et al.*^29^ clarified the impact of cancer therapies on the prevalence of ORN based on 43 articles published between 1990 and 2008. The weighted prevalence for ORN were 7.4%, 5.1%, 6.8% and 5.3% with conventional RT, IMRT, chemoradiotherapy and BT, respectively. Our results show that the mandible is better protected with BT than with VMAT.

Stereotactic radiotherapy with Cyberknife is an attractive option because it delivers a highly conformal dose in a small number of fractions (like BT), with steep dose gradients resulting in reduced normal tissue irradiation and with a short overall treatment time. It can be an efficacious treatment option for recurrent previously irradiated head and neck carcinoma, especially for nonresectable tumours, or in elderly and medically unfit patients. However, in head and neck (oral cavity) tumours with negative lymph node status where definitive local RT is recommended, or in postoperative care where neck RT is not necessary, it has been considered as a therapeutic option, but currently only in the form of clinical trial.^30,31^ The STEREO POSTOP GORTEC 2017-03 trial is a non-randomised phase II trial, the first prospective study to investigate postoperative stereotactic body radiation therapy (SBRT) for head and neck cancers in early-stage oropharyngeal and oral cancers with high-risk surgical margins. In SBRT a total dose of 36 Gy is delivered in 6 fractions over 2 weeks. The primary endpoint is severe late toxicity, with secondary endpoints including acute toxicity, local and locoregional control, disease-free and overall survival, and quality of life, with a planned end date of January 2024.^32^ Stereotactic contouring protocols are very heterogeneous, but generally 1–5 mm for GTV-CTV extension and 2–3 mm for CTV-PTV extension in head and neck cancers.^33^ In our analysis 2 mm for CTV-PTV extension was used.

Zhang Y *et al.*^34^ investigated the feasibility of larynx SBRT therapy planning on a conventional gantry-based linac and compared its plan quality with that made by the Cyberknife on an anthropomorphic head and neck phantom. This study revealed that a gantry-based linear accelerator can achieve similar dosimetric endpoints as the Cyberknife, by employing non-coplanar VMAT arcs.

According to the current study, the CK technic was inferior to BT regarding mandibular Dx (p < 0.001), despite giving better results than VMAT for OARs, while it outperformed BT for the cl. submandibular salivary gland (D_2cm_3: p = 0.0198, D_0.1cm_3: p = 0.0146).

Although the parotid glands are important for salivary secretion, as they provide 70% of the saliva, their relatively large distance from the target volume means that they are not affected by radiation exposure during oral cancer irradiation, which otherwise causes xerostomia.^35^

One of the limitations of our study is that while VMAT and BT techniques are routinely used in the treatment of oral tumours in our department, we have no experience with CK therapy in this setting, the other limitation being that this is a dosimetric comparison without discussing clinical consequences. In the future, it would be interesting to study the side effects and survival parameters when these different radiotherapy modalities are used side by side in the postoperative treatment of oral cavity tumour beds.

## Conclusions

All three irradiation techniques studied resulted in adequate dose distribution in postoperative RT for tongue and floor of mouth cancer. While the doses to small volumes of the mandible was less with BT, in terms of salivary glands, the CK technique resulted in the lowest dose. The highest dose to the critical organs was observed using the VMAT technique. The above results confirm that BT, despite being an invasive technique, is dosimetrically clearly beneficial in the treatment of oral cavity tumours and is a modality worth considering when applying radiotherapy, not only definitively, but also postoperatively. The role of the CK technique for radiotherapy in the head and neck region appears promising, but requires further investigation.
